# Risk of hemorrhagic fever with renal syndrome associated with meteorological factors in diverse epidemic regions: a nationwide longitudinal study in China

**DOI:** 10.1186/s40249-024-01272-7

**Published:** 2025-01-16

**Authors:** Nan Chang, Wenzhong Huang, Yanlin Niu, Zhihu Xu, Yuan Gao, Tingting Ye, Zihao Wang, Xiaohui Wei, Yuming Guo, Qiyong Liu

**Affiliations:** 1 School of Public Health, Nanjing Medical University, Nanjing, Jiangsu China; 2 National Key Laboratory of Intelligent Tracking and Forecasting for Infectious Diseases, National Institute for Communicable Disease Control and Prevention, Chinese Center for Disease Control and Prevention, Beijing, China; 3 Climate, Air Quality Research Unit, School of Public Health and Preventive Medicine, Monash University, Melbourne, Australia; 4 Beijing Center for Disease Prevention and Control, Institute for Nutrition and Food Hygiene, Beijing, China

**Keywords:** Hemorrhagic fever with renal syndrome, Temperature, Humidity, Diverse epidemic regions

## Abstract

**Background:**

Hemorrhagic fever with renal syndrome (HFRS) is a climate-sensitive zoonotic disease that poses a significant public health burden worldwide. While previous studies have established associations between meteorological factors and HFRS incidence, there remains a critical knowledge gap regarding the heterogeneity of these effects across diverse epidemic regions. Addressing this gap is essential for developing region-specific prevention and control strategies. This study conducted a national investigation to examine the associations between meteorological factors and HFRS in three distinct epidemic regions.

**Methods:**

We collected daily meteorological data (temperature and relative humidity) and HFRS incidence cases of 285 cities in China from the Resource and Environment Science and Data Center and the Chinese National Notifiable Infectious Disease Reporting Information System from 2005–2022. Study locations were stratified into three distinct epidemic categories (*Rattus*-dominant, *Apodemus*-dominant, and mixed) based on the seasonality of peak incidence. The associations between meteorological variables and HFRS incidence were investigated using a time-stratified case-crossover design combined with distributed lag nonlinear modeling for each epidemic category.

**Results:**

The exposure-response relationships between meteorological factors and HFRS incidence revealed significant heterogeneity across epidemic regions, as evidenced by Cochran’s Q test for temperature (*Q* = 324.40, *P* < 0.01) and relative humidity (*Q* = 30.57, *P* < 0.01). The optimal daily average temperature for HFRS transmission in *Rattus*-dominant epidemic regions (− 6.6 °C), characterized by spring epidemics, was lower than that observed in *Apodemus*-dominant epidemic regions (13.7 °C), where primary cases occurred during autumn and winter months. Furthermore, the association between relative humidity and HFRS incidence exhibited as a monotonic negative correlation in *Rattus*-dominant regions, while *Apodemus*-dominant regions showed a nonlinear, inverted U-shaped association.

**Conclusions:**

This study highlights the heterogeneous effects of meteorological factors on HFRS incidence across different epidemic regions. Targeted preventive measures should be taken during cold and dry spring days in *Rattus*-dominant regions, and during warm and moderately humid winter days in *Apodemus*-dominant regions. In mixed epidemic regions, both scenarios require attention. These findings provide novel scientific evidence for the formulation and implementation of region-specific HFRS prevention policies.

**Graphical Abstract:**

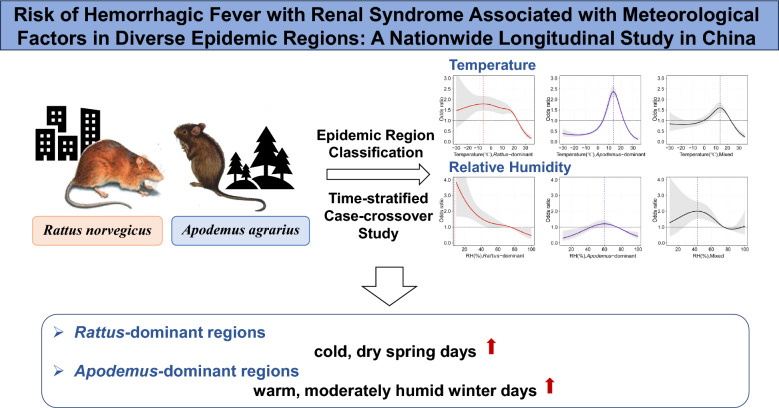

**Supplementary Information:**

The online version contains supplementary material available at 10.1186/s40249-024-01272-7.

## Background

Hemorrhagic fever with renal syndrome (HFRS) is a climate-sensitive zoonotic disease caused by Hantavirus. Transmission to humans occurs through exposure to infectious aerosols generated by contaminated rodents or through the rodent bites [1]. Upon infection, patients typically present with symptoms such as fever, headache, myalgia, kidney damage, and hemorrhagic manifestations [2]. Before the twenty-first century, the annual global number of HFRS patients ranged from approximately 60,000–150,000, with over 90% of cases reported in Asian countries and a fatality rate ranging from 0.1–15% [3, 4]. Although recent advances in vaccines and medical care have reduced mortality rates, many patients continue to experience long-term renal dysfunction or other sequelae following infection [5–7]. Consequently, HFRS remains a significant public health concern.

In China, *Apodemus agrarius* and *Rattus norvegicus* are the primary vectors of HFRS, transmitting Seoul virus (SEOV) and Hantaan virus (HTNV), respectively [8]. Different regions exhibit varying distributions of rodent species, presenting three types of epidemic regions: *Rattus* regions (SEOV), *Apodemus* regions (HTNV), and mixed regions (involving both hosts and virus types). The *Rattus* regions experience peak incidence during spring, whereas the *Apodemus* regions peak in autumn and winter. In mixed regions, two incidence peaks are observed, with the dominant rodent species determining the higher seasonal peak [9].

Extensive epidemiological investigations have established the substantial influence of meteorological factors on HFRS transmission dynamics [10]. However, the existing evidence remains highly inconsistent. Several studies have reported inverse associations between HFRS incidence and antecedent relative humidity (RH) and temperature [11–13], while other investigations have identified significant positive correlations between these meteorological variables and reported HFRS cases [14–16]. Additionally, recent researches [17–19] have revealed that the nonlinear patterns of these associations, with temperature extremes exerting differential effects across various temporal lag periods [20].

Beyond variations in modeling strategies, we attribute the inconsistent findings to the geographic variability of epidemic regions. A national study [21] underscored that epidemic region type is a principal determinant of heterogeneity in the impacts of meteorological factors, suggesting the importance of understanding temperature and humidity variations across different epidemic regions. However, there remains a dearth of understanding regarding the moderating effect of epidemic region types on the association between meteorological factors and HFRS. Additionally, while previous studies [22, 23] have identified gender and age as important factors of HFRS infection, there remains a lack of research on how these demographic factors interact with meteorological variables across different epidemic regions.

Establishing the exposure-response relationships between meteorological factors and HFRS, and comprehensively assessing of the risks in different epidemic regions is crucial for precise prediction and targeted intervention. As China reports the highest number of HFRS cases globally [23], it serves as an ideal setting for conducting HFRS-related research. To address existing research gaps, this study aimed to investigate the associations between meteorological factors and the HFRS incidence risks across different epidemic regions in China, and elucidate the heterogeneous effect patterns of temperature and RH on HFRS. Subgroup analyses stratified by gender and age were performed to identify potentially vulnerable populations within these epidemic regions.

## Methods

### Data collection

The data on daily reported HFRS cases from January 1, 2005 to December 31, 2022 were obtained from the Chinese National Notifiable Infectious Disease Reporting Information System (http://www.chinacdc.cn/) of the Chinese Center for Disease Control and Prevention. This dataset includes information on gender, age, date of onset, and current residence of the reported cases. A total of 202,352 HFRS cases were reported across 393 cities in China. After excluding cases with incomplete address information and cities with fewer than or equal to 1 annual case, we analyzed 202,154 cases from 285 cities in 31 provincial-level administrative divisions (PLADs). These cases accounted for 99.9% of the national cases.

Data on monitoring ambient temperature (℃) and relative humidity (%) within each city was obtained from the Resource and Environment Science and Data Center, Institute of Geographic Science and Natural Resources (https://www.resdc.cn/). City-level daily ambient temperature and relative humidity exposure were calculated as the daily averages of the monitoring values from all stations within each city.

### Epidemic area classification

Evidence from previous literature [21, 24] indicates that the spatial distribution of rodent host species and their associated viruses leads to distinct seasonal patterns. *Apodemus agrarius* transmits HTNV with peak incidence in autumn and winter, while *Rattus norvegicus* carries SEOV with peak incidence occurring in spring. Based on these epidemiological characteristics, we classified all cities into three types of epidemic regions according to the seasonal distribution of HFRS cases. Regions where spring cases (March–July) exceeded 50% of total cases were categorized as *Rattus*-dominant regions, while those with over 50% of cases occurring in autumn–winter (September–January of the following year) were classified as *Apodemus*-dominant regions. The remaining cities, characterized by dual seasonal peaks, were categorized as mixed epidemic regions. To further validate the accuracy of the classification method, we also conducted an extensive review of literature from China that includes rodent surveillance data or virus typing, and cross-referenced the findings from these studies with classification results in the study. The detailed comparisons are presented in Additional file Table S3. In the absence of nationwide distribution data for virus types, seasonal classification of epidemic regions is easier to operate and provides both accessibility and accuracy.

### Statistical analysis

#### Descriptive analysis

The basic characteristics of the study population were described by mean with standard deviation (SD) or interquartile ranges (IQR) for continuous variables, and frequencies for categorical variables. To access group differences, analysis of variance (ANOVA) was conducted for continuous variables and the chi-square test was applied for categorical variables.

#### Exposure-risk analysis

We applied a time-stratified case-crossover design and performed a conditional logistic regression (CLR) with a distributed lag non-linear model (DLNM) [25–28] to examine the associations between ambient temperature and RH with HFRS across different epidemic regions. DLNM enables the simultaneous assessment of non-linear relationships and lagged effects of meteorological exposures on HFRS [29, 30]. Time-stratified case-crossover design inherently controls for time-invariant individual characteristics and time-dependent confounders as each case serves as its own control [31]. By selecting control periods that occur on the same day of the week within the same calendar month, this approach ensures that long-term trends and seasonality effects are eliminated.

The model is expressed as follows:

$$Logit\left( {P\left( {case = I\, in \,stratum_{ij} {|}Temperature, Relative\, Humidity} \right)} \right) = \beta_{ij} + cb\left( {Temperature, df = 4} \right) + cb\left( {Relative\, Humidity,df = 3} \right)$$

In the regression model, ${\beta }_{ij}$ represents the constant term of the stratum. $cb\left(Temperature\right)$ and $cb\left(Relative \,Humidity\right)$ are cross-basis function of daily average temperature and relative humidity. The dependent variable was served to differentiate cases and controls, with “1” denotes the case periods corresponding to HFRS occurrence dates, and “0” represents the control periods, which were selected from the same days of the week within the same calendar month and year for each case. Based on the minimum Akaike Information Criterion (AIC) and Bayesian Information Criterion (BIC), we selected natural cubic splines with 4 and 3 degrees of freedom respectively, to construct the cross-basis functions for temperature and relative humidity. Since the incubation period of HFRS is 1 or 2 weeks, the maximum lag for these factors was set to 60 days [32]. And based on the minimum AIC and BIC, the degrees of freedom for the lag are 3.

We calculated the odds ratio (*OR*) with the 95% confidence interval (*CI*) for risk inference. Based on our initial analysis, we selected 23 °C and 75% relative humidity as reference points, as they represent comfortable environmental conditions relevant to human activities. The 5th and 95th percentiles of temperature and relative humidity were adopted to represent the lag-effect relationship between meteorological factors and HFRS under extreme weather conditions. Specifically, these percentiles correspond to high temperature (28.8 °C), low temperature (− 7.3 °C), high relative humidity (91.8%), and low relative humidity (35.2%).

To investigate the potential modification effect, stratified analyses were conducted by gender and age groups. The age categories were established as 0–35 years, 35–65 years, and ≥ 65 years, based on previous literature [33–35] and the demographic distribution of HFRS cases in China, represented young people, middle-aged adults, and senior citizens, respectively. We applied simple meta-analysis and the Cochran Q test to examine the heterogeneity between subgroups.

#### Sensitivity analysis

Sensitivity analyses were performed by adjusting the parameters of the CLR model to assess the robustness of our findings [36]. First, cities were categorized into five groups based on their epidemic patterns: *Rattus*-only regions (≥ 70% cases occurring from March–July), *Apodemus*-only regions (≥ 70% cases occurring from September–January of the following year), *Rattus*-dominant regions (50–70% cases occurring from March–July), *Apodemus*-dominant regions (50–70% cases occurring from September–January of the following year), and mixed epidemic regions (all other patterns). We also altered the maximum lag of daily mean temperature from 60 to 45 days and 75 days. To ensure that the heterogeneity across different epidemic regions is not due to variations in temperature distribution, we included the average temperature and average RH of each area as predictors in a multivariate regression model [37]. Additionally, wind speed, precipitation, and sunshine duration were included as covariates in the model to ensure that these meteorological factors did not influence the associations between temperature, humidity, and the incidence of HFRS.

All analyses were performed with the statistical software R 4.3.1 (R Foundation for Statistical Computing, Vienna, Austria). “dlnm”, “survival”, and “mixmeta” packages were used for modeling and the *P* < 0.05 was considered statistically significant. ArcGIS 10.5 (ESRI Inc., Redlands, CA, USA) was used for the calculation of epidemiological data.

## Results

### Descriptive results

**Fig. 1:**
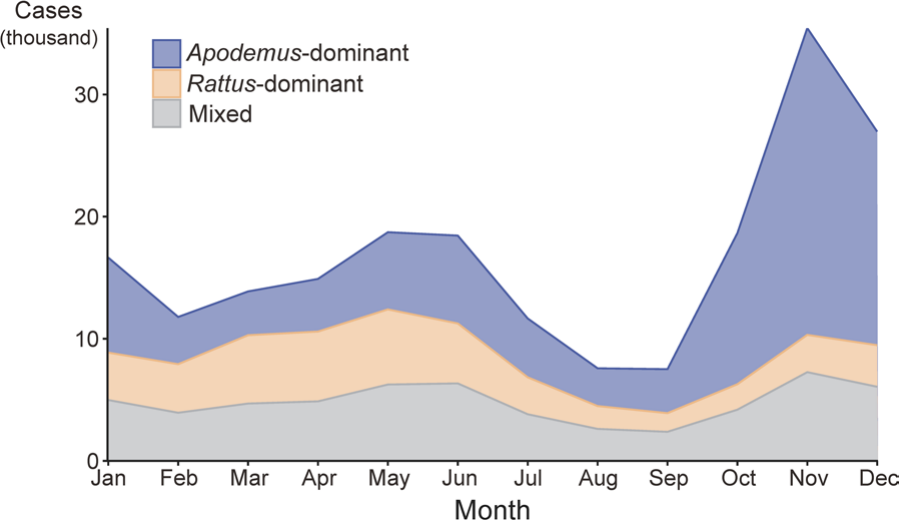
Monthly distribution of HFRS cases in different epidemic regions in China, 2005−2022

A total of 202,154 HFRS cases were analyzed from 285 cities in 31 PLADs. The epidemic regions comprised 83 *Rattus*-dominant cities (29.1%), 114 *Apodemus*-dominant cities (40.0%), and 88 mixed cities (30.9%). Detailed epidemic area information for each city is provided in Additional file Table S1. Regarding epidemic seasonality, *Rattus*-dominant epidemic regions exhibited peak HFRS incidence during spring, whereas *Apodemus*-dominant epidemic regions experienced their highest case counts in winter (Fig. 1). Males and middle-aged adults were the primary groups affected by HFRS. Compared to other epidemic regions, *Rattus*-dominant regions had a higher proportion of women and younger individuals, while *Apodemus*-dominant regions had a greater proportion of elderly patients (Table 1). There were significant differences in the distribution of gender (*χ*^*2*^ = 21.7, *P* < 0.01) and age groups (*χ*^*2*^ = 298.3, *P* < 0.01) among the epidemic regions according to the chi-square test.

**Table 1:** Characteristics of HFRS cases and distribution of climatic factors in three types of epidemic regions in China, 2005–2022

	*Rattus*-dominant area	*Apodemus*-dominant area	Mixed area	Overall
	(*n* = 45,202)	(*n* = 99,512)	(*n* = 57,440)	(*n* = 202,154)
Gender (%)^*^				
Female	11,946 (26.4)	25,176 (25.3)	14,616 (25.4)	51,738 (25.6)
Male	33,256 (73.6)	74,336 (74.7)	42,824 (74.6)	150,416 (74.4)
Age, years (%)*				
0–35	13,091 (29.0)	26,242 (26.4)	16,371 (28.5)	55,704 (27.6)
35–65	28,189 (62.4)	62,593 (62.9)	35,963 (62.6)	126,745 (62.7)
≥ 65	3922 (8.7)	10,677 (10.7)	5106 (8.9)	19,705 (9.7)
Temp (℃)*				
Min	− 30	− 38.8	− 32.1	− 38.8
Median	17.6	15.1	17.5	16.6
Max	37.1	36.8	36.5	37.1
Mean	14.9	13.7	15.5	14.6
SD	11.4	11.1	10.7	11.1
RH (%)*				
Min	8.4	8.8	7.3	7.3
Median	71.3	72.2	73.8	72.5
Max	100	100	100	100
Mean	67.9	70.1	71.3	69.8
SD	17.5	15.6	15.4	16.2

*Temp* temperature, *RH* relative humidity, *SD* standard deviation

^*^Statistically significant difference among different epidemic regions (*P* ≤ 0.05)

In *Apodemus*-dominant regions, the daily average temperature was lower compared to *Rattus*-dominant regions, while the relative humidity was higher. Specific details are presented in Table 1. Results of the ANOVA test indicated significant differences in the distribution of daily average temperature (*F* = 5049, *P* < 0.01) and relative humidity (*F* = 6189, *P* < 0.01) among the three epidemic region types.

### Association between meteorological factors and HFRS

**Fig. 2:**
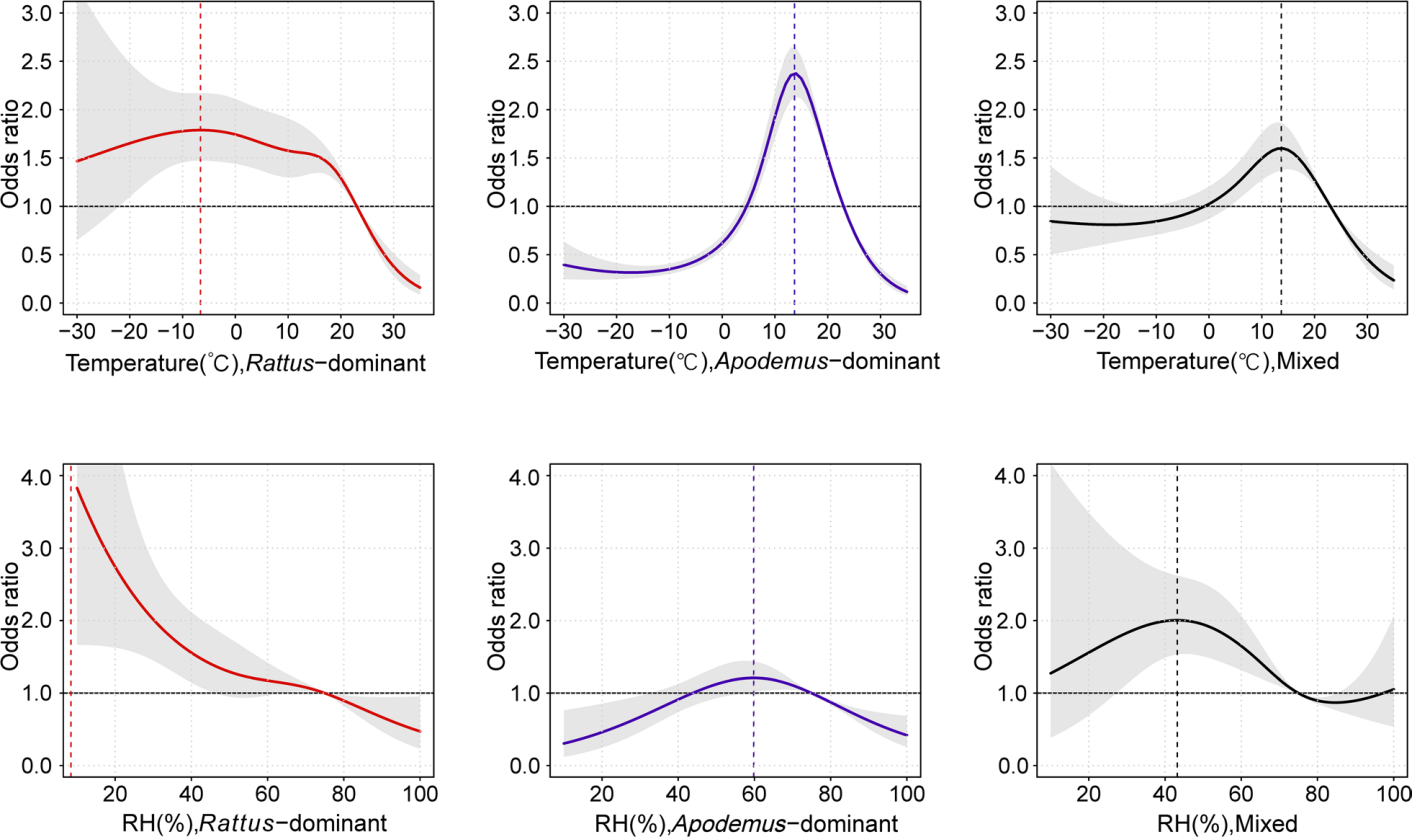
The cumulative odds ratio of HFRS in three types of epidemic regions over the 60 days after the exposure. *RH* relative humidity. Notes: The reference point for temperature is 23℃, for relative humidity is 75%

The cumulative effects of temperature and relative humidity on HFRS over a 60-day lag period are illustrated in Fig.2. In *Rattus*-dominant regions, the highest risk temperature was − 6.6 °C, and the incidence risk of HFRS decreased gradually with increasing relative humidity. In *Apodemus*-dominant and mixed epidemic regions, the highest risk occurred at 13.7 °C. The relationship between relative humidity and HFRS followed an inverted U-shaped curve, with the peak risk observed at 59.8% relative humidity in *Apodemus*-dominant regions and at 43.3% in mixed epidemic regions. The results of Cochran’s Q test revealed significant differences in the exposure–response relationships between meteorological factors (temperature:* Q* = 324.40, *P* < 0.01; relative humidity: *Q* = 30.57, *P* < 0.01) and HFRS among the three epidemic region types. The optimal temperatures for HFRS transmission were higher in *Apodemus*-dominant and mixed epidemic regions compared to *Rattus*-dominant regions.

**Fig. 3:**
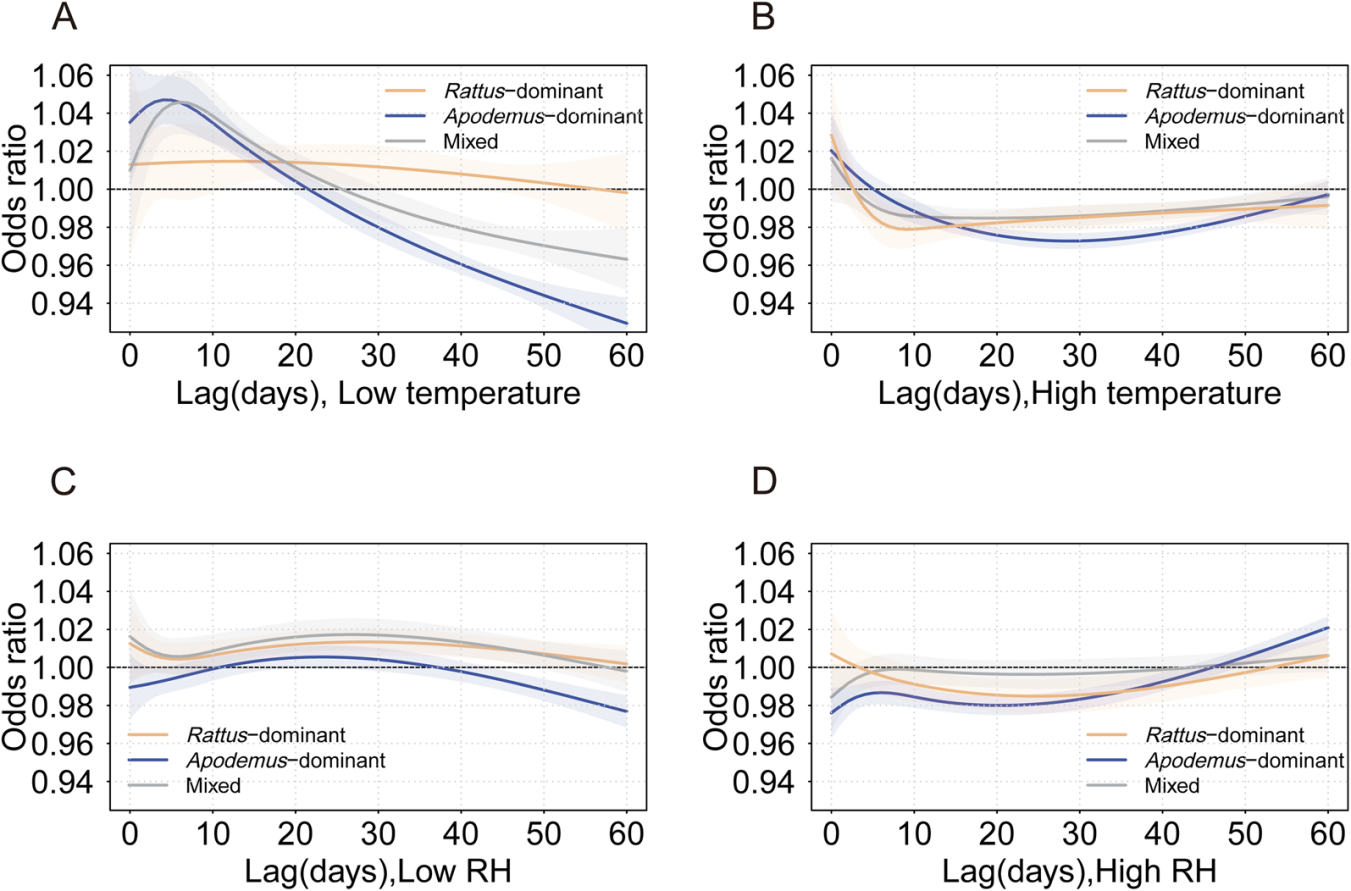
The odds ratio of HFRS associated with low temperature, high temperature, low RH and high RH during the 60 days after the exposure. *RH* relative humidity. Notes: Low temperature, -7.3℃; High temperature, 28.8℃; Low RH: 35.2%; High RH: 91.8%. The reference point for temperature is 23℃, for relative humidity is 75%

Figure 3 shows the lag-response curves for different epidemic regions over a 0–60 day under low temperature (− 7.3 °C), high temperature (28.8 °C), low relative humidity (35.2%), and high relative humidity (91.8%) conditions. Low temperatures acted as a relatively stable risk-promoting factor in *Rattus*-dominant regions, and exhibited a promotive effect on HFRS cases but transitioned into an increasingly inhibitory effect over time in *Apodemus*-dominant regions. High temperature significantly inhibited HFRS across all regions, and an initial decline in risk was observed, stabilizing after approximately 30 days. Under low RH conditions, the risk in *Rattus*-dominant regions remained consistently higher compared to *Apodemus*-dominant and mixed regions, which both showed slight fluctuations. Under high RH conditions, relative humidity had a suppressive effect on HFRS prevalence of HFRS in both *Rattus*-dominant and *Apodemus*-dominant regions during the 10–50 days lag period.

The association between meteorological factors and HFRS varied by gender and age groups. Multivariate Cochran Q-test revealed significant effect heterogeneity of gender on RH in *Rattus*-dominant area (*Q* = 7.6, *P* = 0.04), and of age on temperature in *Apodemus*-dominant area (*Q* = 29.9, *P* < 0.01). No significant effect modifications were observed in other groups (*P* > 0.05). In *Rattus*-dominant regions, a substantial gender-based variation was observed in RH effects, with low RH conditions exhibiting a stronger inhibitory effect in females compared to males. In *Apodemus*-dominant regions, low temperature conditions exerted a weaker inhibitory effect on young individuals, whereas high temperature conditions exerted a weaker inhibitory effect on senior citizens (Additional file Table S2).

### Sensitivity analysis results

The results of all sensitivity analyses confirmed the robustness of our main finding, demonstrating that our conclusions remained stable regardless of variations in the classification of epidemic regions (Additional file Figure S1), different lag days (Additional file Figure S2–S3), or the inclusion of additional meteorological factors as covariates (Additional file Figure S4). When utilizing the five-category classification approach, the *Rattus*-only regions had a small number of cases, leading to overly wide confidence intervals and rendering the results less reliable. The cumulative effect curve of *Rattus*-dominant regions was similar in both the five-category and three-category classification approaches, as were the cumulative effect curves of *Apodemus*-only and *Apodemus*-dominant regions in the five-category method compared to the *Apodemus*-dominant regions in the three-category method. The mixed epidemic regions remained consistent across both classification methods. These similarities can be observed by comparing the cumulative effect curves in Additional file Figure S1 with the trends presented in Fig. 2 of the main text. Similarly, the cumulative effect curves under different lag days showed consistent trends across the models. This trend stability is evident when comparing the lag-specific cumulative effect curves presented in Additional file Figure S2–S3 with those in Fig. 2 of the main text, further supporting the robustness of our findings. The inclusion of additional meteorological covariates (wind speed, precipitation, and hours of sunshine) modeled using natural cubic splines (ns) was tested as part of the sensitivity analyses. Separate models were developed for each of the three epidemic regions, with one model adjusted for these additional factors and another unadjusted. Likelihood ratio tests using ANOVA showed no significant differences between the adjusted and unadjusted models in any epidemic region (*Rattus*-dominant regions: *χ*^*2*^ = 9.2, *P* = 0.42; *Apodemus*-dominant regions: *χ*^*2*^ = 12.6, *P* = 0.18; mixed regions: *χ*^*2*^ = 5.9, *P* = 0.75), confirming that the addition of these covariates did not substantially affect the results.

## Discussion

This study utilized the national surveillance data of HFRS in China during 2005–2022 to establish a time-stratified case-control model. To the best of our knowledge, this is the first nationwide study to investigate the impacts of temperature and RH on the prevalence of HFRS in different epidemic regions at a daily timescale. The results showed significant differences in the association between climate factors and the number of HFRS cases among different epidemic regions. In *Rattus*-dominant regions, the optimal temperature for transmission was lower than that in *Apodemus*-dominant regions. The risk of HFRS was higher in *Rattus*-dominant regions but lower in *Apodemus*-dominant regions under low relative humidity. The incidence risk of HFRS was found to be more strongly influenced by ambient relative humidity in *Rattus*-dominant regions, whereas temperature had a greater impact in *Apodemus*-dominant regions.

Our findings provide evidence of the exposure-response relationship between meteorological factors and HFRS across different epidemic regions, aligning with previous studies conducted in specific epidemic regions [38–40]. In *Rattus*-dominant regions, HFRS cases increased under dry and cold condition. The prolonged survival time of SEOV under low-temperature conditions might enhance environmental virus stability and infectivity, thereby contributing to the elevated risk of HFRS [19]. Moreover, dry and cold conditions may influence rodent behavior and human activities, increasing the likelihood of human exposure and disease transmission during these periods [38]. In *Apodemus*-dominant regions, both temperature and relative humidity exhibited an inverted U-shaped relationship with HFRS, consistent with earlier findings [20]. Specifically, within the range of 0–60% relative humidity, higher humidity was associated with increased HTNV risk. One possible explanation is that moderate humidity enhances HTNV stability and infectivity in the environment, increasing the risk of human exposure. Humid conditions also support *Apodemus agrarius* survival and reproduction, leading to higher rodent densities and greater human contact. In contrast, excessively high humidity may reduce rodent populations by limiting habitat suitability and food availability, which in turn lowers the risk of HFRS transmission [41].

We also found that the optimal temperature for *Rattus*-dominant regions, where primary cases occurred in spring, was higher than in *Apodemus*-dominant regions, where primary cases occurred in the autumn and winter. This discrepancy may primarily be attributed to differences in the dominant rodent species. In *Rattus*-dominant regions, the primary reservoir hosts exhibit a marked preference for habitats near human settlements. During the cold spring days, these rodents are more likely to invade human living spaces, increasing the likelihood of human-rodent interactions. Additionally, low ambient temperatures are often associated with reduced ventilation in human dwellings, which may facilitate the transmission of aerosolized viral particles [42]. Conversely, the survival rate of *Apodemus agrarius*, the dominant wild rodent in *Apodemus*-dominant regions, tends to increase during warm winters [43]. This higher survival rate likely leads to an increased density of reservoir hosts, thereby elevating the risk of infection among human populations.

In mixed epidemic regions, where both rodent species and their associated Hantavirus strains coexist, meteorological influences exhibit composite patterns that reflect the epidemiological characteristics of both species. These regions require comprehensive surveillance strategies that address the spring-associated risks observed in *Rattus*-dominant regions and the winter-associated patterns typical of *Apodemus*-dominant regions. This dual vulnerability necessitates the need of year-round vigilance and adaptive prevention strategies to effectively mitigate transmission risks across multiple seasonal peaks.

In the lag-effect relationship, we found that low temperatures led to a short-term increase in HFRS cases in *Apodemus*-dominant regions. Although the underlying mechanism remains unclear, considering the length of the incubation period, we speculate that low temperatures may prompt more HFRS patients to seek medical treatment. We also observed that in *Rattus*-dominant regions, humid environments promoted HFRS over a longer lag period, which was consistent with earlier studies [44]. Conversely, low relative humidity exhibited an inhibitory effect over a longer lag period, which may be related to vegetation growth [45]. The short-term inhibitory effect of high relative humidity may be due to the disruption of the microenvironment or changes in rodent population activity, negatively impacting disease transmission [46]. Our results further showed that mixed epidemic regions closely resembled the *Apodemus*-dominant regions, suggesting that meteorological factors have a greater impact on HTNV, aligning with previous findings [24].

Gender and age significantly modified the effects of RH in *Rattus*-dominant regions and temperature in *Apodemus*-dominant regions, respectively. These differences may be attributed to the environment and modes of contact with infectious sources among different populations and vaccination coverage in each epidemic area [47, 48]. The underlying mechanisms of the modification effect remain unclear.

Based on our findings, we recommend implementing timely and effective preventive measures under specific meteorological conditions. In *Rattus*-dominant regions, during cold and dry spring weather, and in *Apodemus*-dominant regions, during warm winters, collective vaccination programs should be conducted for susceptible populations. Additionally, rodent habitats should be cleaned, and rodent control measures, such as placing glue boards and traps both indoors and outdoors, should be deployed to minimize human-rodent contact.

This study is the first nationwide study in China to utilize fine-scale (daily) exposure data to examine the exposure-response relationships between meteorological factors and HFRS across different epidemic regions. The time-stratified case-crossover design offers methodological advantages, including independence from sample size and inherent control of temporal confounders, effectively accounting for both long-term and seasonal trends [26]. Our classification method for epidemic regions is simple, feasible, and highly generalizable. The main findings provide valuable guidance for cities to implement timely measures, such as rodent control and vaccination, which are crucial for the prevention of HFRS and the allocation of health resources.

Some limitations should be noted. First, residual confounding cannot be excluded due to the observational nature of the studies. Second, subclinical infections were not considered, although prior research has shown that the rate of subclinical infection does not exceed 5% [49].

## Conclusions

The associations between meteorological parameters and HFRS incidence demonstrate significant heterogeneity across distinct epidemic regions. In *Rattus*-dominant regions, enhanced surveillance and preventive measures should be prioritized during cold, dry spring conditions, whereas in *Apodemus*-dominant regions, vigilance should be heightened during periods of moderate humidity and elevated temperatures in the winter months. For mixed-epidemic regions, comprehensive monitoring strategies addressing both scenarios are warranted to effectively mitigate HFRS transmission risk through timely implementation of preventive interventions.

## Supplementary Information

Additional file 1

## Data Availability

The datasets used and analyzed during the current study are available from the corresponding author on reasonable request.

## Abbreviations

HFRS: Hemorrhagic fever with renal syndrome
SEOV: Seoul virus
HTNV: Hantaan virus
SD: Standard deviation
IQR: Interquartile ranges
ANOVA: Analysis of variance
CLR: Conditional logistic regression
DLNM: Distributed lag non-linear model
AIC: Akaike Information Criterion
BIC: Bayesian Information Criterion
OR: Odds ratio
*CI*: Confidence Interval
RH: Relative humidity
IQR: Interquartile ranges
