# Supplementary material for: Risk of hemorrhagic fever with renal syndrome associated with meteorological factors in diverse epidemic regions: a nationwide longitudinal study in China
Source: Infect Dis Poverty. 2025 Jan 16;14:3. doi: 10.1186/s40249-024-01272-7 (PMC11737169; doi:10.1186/s40249-024-01272-7)
Supplement: Supplementary file 1 — Additional file 1 [file 40249_2024_1272_MOESM1_ESM.docx]

**Additional file**

**Risk of Hemorrhagic Fever with Renal Syndrome Associated with Meteorological Factors in Diverse Epidemic Regions: A Nationwide Longitudinal Study in China**

Nan Chang, Wenzhong Huang, Yanlin Niu, Zhihu Xu, Yuan Gao, Tingting Ye, Zihao Wang, Xiaohui Wei, Yuming Guo^*^ and Qiyong Liu^*^

**Table S1.** City-level classification of epidemic regions for hemorrhagic fever with renal syndrome cases in China, 2005-2022.

**Table S2.** The odds ratio of HFRS associated with low temperature, high temperature, low RH and high RH in gender and age subgroups

**Figure S1.** The cumulative odds ratio of HFRS in five types of epidemic areas over the 60 days after the exposure

**Figure S2.** The cumulative odds ratio of HFRS in three types of epidemic areas over the 45 days after the exposure

**Figure S3.** The cumulative odds ratio of HFRS in three types of epidemic areas over the 75 days after the exposure

**Figure S4.** The cumulative odds ratio of HFRS in three types of epidemic areas over the 60 days after exposure, adjusted for additional meteorological covariates (wind speed, precipitation, and hours of sunshine)

**Table S3.** Comparison of Literature-Based Classification Results with Study's Classification

**Table S1** City-level classification of epidemic regions for hemorrhagic fever with renal syndrome cases in China, 2005-2022.

| City ID | City | Classification^*^ |
| --- | --- | --- |
| 110000 | Beijing | A |
| 120000 | Tianjin | C |
| 130100 | Shijiazhuang | A |
| 130200 | Tangshan | A |
| 130300 | Qinhuangdao | A |
| 130400 | Handan | C |
| 130500 | Xingtai | A |
| 130600 | Baoding | A |
| 130700 | Zhangjiakou | A |
| 130800 | Chengde | A |
| 130900 | Cangzhou | A |
| 131000 | Langfang | C |
| 131100 | Hengshui | A |
| 140100 | Taiyuan | A |
| 140200 | Datong | C |
| 140300 | Yangquan | C |
| 140400 | Changzhi | C |
| 140500 | Jinzhong | B |
| 140600 | Shuozhou | A |
| 140700 | Jincheng | A |
| 140800 | Yuncheng | B |
| 140900 | Xinzhou | A |
| 141000 | Linfen | C |
| 141100 | Lüliang | C |
| 150100 | Hohhot | A |
| 150200 | Baotou | A |
| 150400 | Chifeng | A |
| 150500 | Tongliao | A |
| 150600 | Ordos | C |
| 150700 | Hulunbuir | B |
| 150800 | Bayannur | A |
| 150900 | Ulanqab | A |
| 152200 | Xing'an League | B |
| 152500 | Xilingol League | C |
| 210100 | Shenyang | A |
| 210200 | Dalian | A |
| 210300 | Anshan | C |
| 210400 | Fushun | B |
| 210500 | Benxi | C |
| 210600 | Dandong | B |
| 210700 | Jinzhou | A |
| 210800 | Yingkou | A |
| 210900 | Fuxin | A |
| 211000 | Liaoyang | B |
| 211100 | Panjin | A |
| 211200 | Tieling | B |
| 211300 | Chaoyang | A |
| 211400 | Huludao | A |
| 220100 | Changchun | A |
| 220200 | Jilin | A |
| 220300 | Siping | A |
| 220400 | Liaoyuan | C |
| 220500 | Tonghua | C |
| 220600 | Baishan | C |
| 220700 | Songyuan | A |
| 220800 | Baicheng | A |
| 222400 | Yanbian Korean Autonomous Prefecture | B |
| 230100 | Harbin | C |
| 230200 | Qiqihar | B |
| 230300 | Jixi | B |
| 230400 | Hegang | B |
| 230500 | Shuangyashan | B |
| 230600 | Daqing | A |
| 230700 | Yichun | B |
| 230800 | Jiamusi | C |
| 230900 | Qitaihe | B |
| 231000 | Mudanjiang | C |
| 231100 | Heihe | B |
| 231200 | Suhua | C |
| 232700 | Daxinganling | B |
| 310000 | Shanghai | C |
| 320100 | Nanjing | A |
| 320200 | Wuxi | C |
| 320300 | Xuzhou | B |
| 320400 | Changzhou | A |
| 320500 | Suzhou | A |
| 320600 | Nantong | A |
| 320700 | Lianyungang | B |
| 320800 | Huaian | B |
| 320900 | Yancheng | B |
| 321000 | Yangzhou | B |
| 321100 | Zhenjiang | A |
| 321200 | Taizhou | B |
| 321300 | Suqian | B |
| 330100 | Hangzhou | C |
| 330200 | Ningbo | A |
| 330300 | Wenzhou | C |
| 330400 | Jiaxing | B |
| 330500 | Huzhou | A |
| 330600 | Shaoxing | C |
| 330700 | Jinhua | B |
| 330800 | Quzhou | B |
| 330900 | Zhoushan | C |
| 331000 | Taizhou | B |
| 331100 | Lishui | B |
| 340100 | Hefei | C |
| 340200 | Wuhu | A |
| 340300 | Bengbu | B |
| 340400 | Huainan | B |
| 340500 | Ma'anshan | C |
| 340600 | Huaibei | B |
| 340700 | Tongling | C |
| 340800 | Anqing | A |
| 341000 | Huangshan | C |
| 341100 | Chuzhou | B |
| 341200 | Fuyang | B |
| 341300 | Suzhou | B |
| 341500 | Lu'an | B |
| 341600 | Bozhou | B |
| 341700 | Chizhou | B |
| 341800 | Xuancheng | A |
| 350100 | Fuzhou | A |
| 350200 | Xiamen | A |
| 350300 | Putian | C |
| 350400 | Sanming | A |
| 350500 | Quanzhou | A |
| 350600 | Zhangzhou | C |
| 350700 | Nanping | C |
| 350800 | Longyan | C |
| 350900 | Ningde | C |
| 360100 | Nanchang | C |
| 360200 | Jingdezhen | A |
| 360300 | Pingxiang | C |
| 360400 | Jiujiang | B |
| 360500 | Xinyu | B |
| 360600 | Yingtan | C |
| 360700 | Ganzhou | A |
| 360800 | Ji'an | B |
| 360900 | Yichun | B |
| 361000 | Fuzhou | B |
| 361100 | Shangrao | C |
| 370100 | Jinan | C |
| 370200 | Qingdao | B |
| 370300 | Zibo | C |
| 370400 | Zaozhuang | B |
| 370500 | Dongying | C |
| 370600 | Yantai | B |
| 370700 | Weifang | B |
| 370800 | Jining | C |
| 370900 | Tai'an | C |
| 371000 | Weihai | C |
| 371100 | Rizhao | B |
| 371300 | Linyi | B |
| 371400 | Dezhou | C |
| 371500 | Liaocheng | B |
| 371600 | Binzhou | C |
| 371700 | Heze | C |
| 410100 | Zhengzhou | B |
| 410200 | Kaifeng | B |
| 410300 | Luoyang | B |
| 410400 | Pingdingshan | B |
| 410500 | Anyang | B |
| 410600 | Hebi | C |
| 410700 | Xinxiang | B |
| 410800 | Jiaozuo | A |
| 410900 | Puyang | B |
| 411000 | Xuchang | B |
| 411100 | Luohe | B |
| 411200 | Sanmenxia | B |
| 411300 | Nanyang | B |
| 411400 | Shangqiu | B |
| 411500 | Xinyang | B |
| 411600 | Zhoukou | B |
| 411700 | Zhumadian | B |
| 419001 | Jiyuan | B |
| 420100 | Wuhan | C |
| 420200 | Huangshi | B |
| 420300 | Shiyan | B |
| 420500 | Yichang | A |
| 420600 | Xiangyang | C |
| 420700 | Ezhou | C |
| 420800 | Jingmen | B |
| 420900 | Xiaogan | C |
| 421000 | Jingzhou | A |
| 421100 | Huanggang | A |
| 421200 | Xianning | C |
| 421300 | Suizhou | A |
| 422800 | Enshi Tujiaand Miao Autonomous Prefecture | B |
| 429004 | Xiantao | C |
| 429005 | Qianjiang | C |
| 430100 | Changsha | C |
| 430200 | Zhuzhou | B |
| 430300 | Xiangtan | C |
| 430400 | Hengyang | B |
| 430500 | Shaoyang | C |
| 430600 | Yueyang | C |
| 430700 | Changde | C |
| 430800 | Zhangjiajie | B |
| 430900 | Yiyang | A |
| 431000 | Chenzhou | B |
| 431100 | Yongzhou | B |
| 431200 | Huaihua | B |
| 431300 | Loudi | C |
| 433100 | Xiangxi Tujiaand Miao Autonomous Prefecture | B |
| 440100 | Guangzhou | C |
| 440200 | Shaoguan | C |
| 440300 | Shenzhen | C |
| 440400 | Zhuhai | A |
| 440500 | Shantou | A |
| 440600 | Foshan | C |
| 440700 | Jiangmen | A |
| 440800 | Zhanjiang | A |
| 440900 | Maoming | C |
| 441200 | Zhaoqing | C |
| 441300 | Huizhou | A |
| 441400 | Meizhou | A |
| 441500 | Shanwei | C |
| 441600 | Heyuan | A |
| 441700 | Yangjiang | A |
| 441800 | Qingyuan | C |
| 441900 | Dongguan | C |
| 442000 | Zhongshan | A |
| 445100 | Chaozhou | A |
| 445200 | Jieyang | A |
| 445300 | Yunfu | A |
| 450100 | Nanning | A |
| 450300 | Guilin | B |
| 450400 | Wuzhou | A |
| 450700 | Qinzhou | B |
| 450800 | Guigang | C |
| 450900 | Yulin | C |
| 451100 | Hezhou | A |
| 460100 | Haikou | A |
| 469003 | Danzhou | C |
| 500000 | Chongqing | C |
| 510100 | Chengdu | B |
| 510300 | Zigong | A |
| 510400 | Panzhihua | A |
| 510500 | Luzhou | C |
| 510600 | Deyang | A |
| 510700 | Mianyang | B |
| 510800 | Guangyuan | B |
| 510900 | Suining | B |
| 511000 | Neijiang | A |
| 511100 | Leshan | C |
| 511300 | Nanchong | B |
| 511400 | Meishan | B |
| 511500 | Yibin | B |
| 511600 | Guang'an | B |
| 511700 | Dazhou | B |
| 511900 | Bazhong | B |
| 512000 | Ziyang | B |
| 513300 | Ganzi Tibetan Autonomous Prefecture | B |
| 513400 | Liangshan Yi Autonomous Prefecture | C |
| 520100 | Guiyang | B |
| 520200 | Liupanshui | A |
| 520300 | Zunyi | C |
| 520500 | Bijie | C |
| 520600 | Tongren | C |
| 522300 | Qiannan Buyeiand Miao Autonomous Prefecture | C |
| 522600 | Qiandongnan Miaoand Dong Autonomous Prefecture | B |
| 522700 | Qiannan Buyeiand Miao Autonomous Prefecture | C |
| 530100 | Kunming | C |
| 530300 | Qujing | C |
| 530400 | Yuxi | C |
| 530500 | Baoshan | B |
| 530600 | Zhaotong | A |
| 530700 | Lijiang | A |
| 532300 | Chuxiong Yi Autonomous Prefecture | A |
| 532500 | Honghe Haniand Yi Autonomous Prefecture | C |
| 532600 | Wenshan Zhuangand Miao Autonomous Prefecture | A |
| 532900 | Dali Bai Autonomous Prefecture | A |
| 533300 | Nujiang Lisu Autonomous Prefecture | C |
| 533400 | Diqing Tibetan Autonomous Prefecture | C |
| 540300 | Changdu | B |
| 610100 | Xi'an | B |
| 610200 | Tongchuan | B |
| 610300 | Baoji | B |
| 610400 | Xianyang | B |
| 610500 | Weinan | B |
| 610600 | Yan'an | B |
| 610700 | Hanzhong | B |
| 610800 | Yulin | B |
| 610900 | Ankang | B |
| 611000 | Shangluo | B |
| 620100 | Lanzhou | B |
| 620500 | Tianshui | B |
| 620800 | Pingliang | B |
| 621000 | Qingyang | B |
| 621100 | Dingxi | B |
| 621200 | Longnan | B |
| 623000 | Gannan Tibetan Autonomous Prefecture | B |
| 630100 | Xining | C |
| 630200 | Haidong | B |
| 640400 | Guyuan | B |
| 653100 | Kashgar Prefecture | A |

^*^A: *Rattus*-dominant area; B: *Apodemus*-dominant area; C: mixed epidemic area

**Table S2** The odds ratio of HFRS associated with low temperature, high temperature, low RH and high RH in gender and age subgroups.

|  |  | Gender [*OR*(95%*CI*)] | | | Age, years[*OR*(95%*CI*)] | | | |
| --- | --- | --- | --- | --- | --- | --- | --- | --- |
|  |  | Male^a^ | Female^a^ | *P* | <35^a^ | 35-65^a^ | ≥65^a^ | *P* |
| *Rattus*-  dominant | Low temp | 1.70(1.36,2.13) | 2.06(1.41,3.00) | 0.39352 | 2.16(1.49,3.15) | 1.80(1.41,2.29) | 1.07(0.56,2.06) | 0.186913 |
|  | High temp | 0.49(0.37,0.63) | 0.39(0.25,0.63) | 0.44823 | 0.46(0.29,0.73) | 0.44(0.32,0.58) | 0.64(0.32,1.31) | 0.608451 |
|  | Low RH | 2.09(1.47,2.96) | 1.06(0.59,1.91) | 0.05255 | 1.45(0.82,2.59) | 1.81(1.24,2.65) | 2.06(0.78,5.44) | 0.767455 |
|  | High RH | 0.81(0.50,1.30) | 0.32(0.15,0.70) | 0.04979* | 0.55(0.24,1.26) | 0.60(0.36,1.00) | 0.97(0.29,3.17) | 0.727873 |
| *Apodemus*-  dominant | Low temp | 0.37(0.32,0.44) | 0.44(0.33,0.57) | 0.32704 | 0.65(0.49,0.85) | 0.35(0.29,0.41) | 0.23(0.15,0.35) | 0.000020* |
|  | High temp | 0.38(0.32,0.45) | 0.38(0.28,0.51) | 0.99155 | 0.39(0.29,0.53) | 0.34(0.28,0.41) | 0.61(0.40,0.93) | 0.042341* |
|  | Low RH | 0.79(0.58,1.07) | 0.82(0.48,1.39) | 0.90266 | 0.93(0.56,1.55) | 0.80(0.57,1.12) | 0.59(0.26,1.32) | 0.644379 |
|  | High RH | 0.62(0.47,0.82) | 0.48(0.30,0.78) | 0.36495 | 0.82(0.5,1.35) | 0.55(0.40,0.74) | 0.42(0.22,0.81) | 0.233888 |
| Mixed | Low temp | 0.93(0.76,1.13) | 0.72(0.51,1.01) | 0.19631 | 1.17(0.84,1.63) | 0.82(0.66,1.01) | 0.51(0.28,0.92) | 0.037877* |
|  | High temp | 0.56(0.46,0.70) | 0.45(0.32,0.64) | 0.27951 | 0.59(0.41,0.87) | 0.48(0.38,0.61) | 0.61(0.37,1.01) | 0.537958 |
|  | Low RH | 2.32(1.53,3.52) | 1.07(0.51,2.22) | 0.0712 | 3.08(1.56,6.07) | 1.84(1.16,2.92) | 0.71(0.21,2.38) | 0.107402 |
|  | High RH | 0.91(0.61,1.35) | 0.93(0.48,1.79) | 0.95005 | 1.31(0.64,2.68) | 0.94(0.61,1.43) | 0.41(0.16,1.05) | 0.152767 |

^a^*OR*(95% *CI*): The reference point for temperature is 23℃, for relative humidity is 75%.

Abbreviations: *Temp* temperature; *RH* relative humidity; *OR* odds rate; *CI* confidence interval.

^*^Statistically significant difference among different subgroups (*P* ≤ 0.05)

**
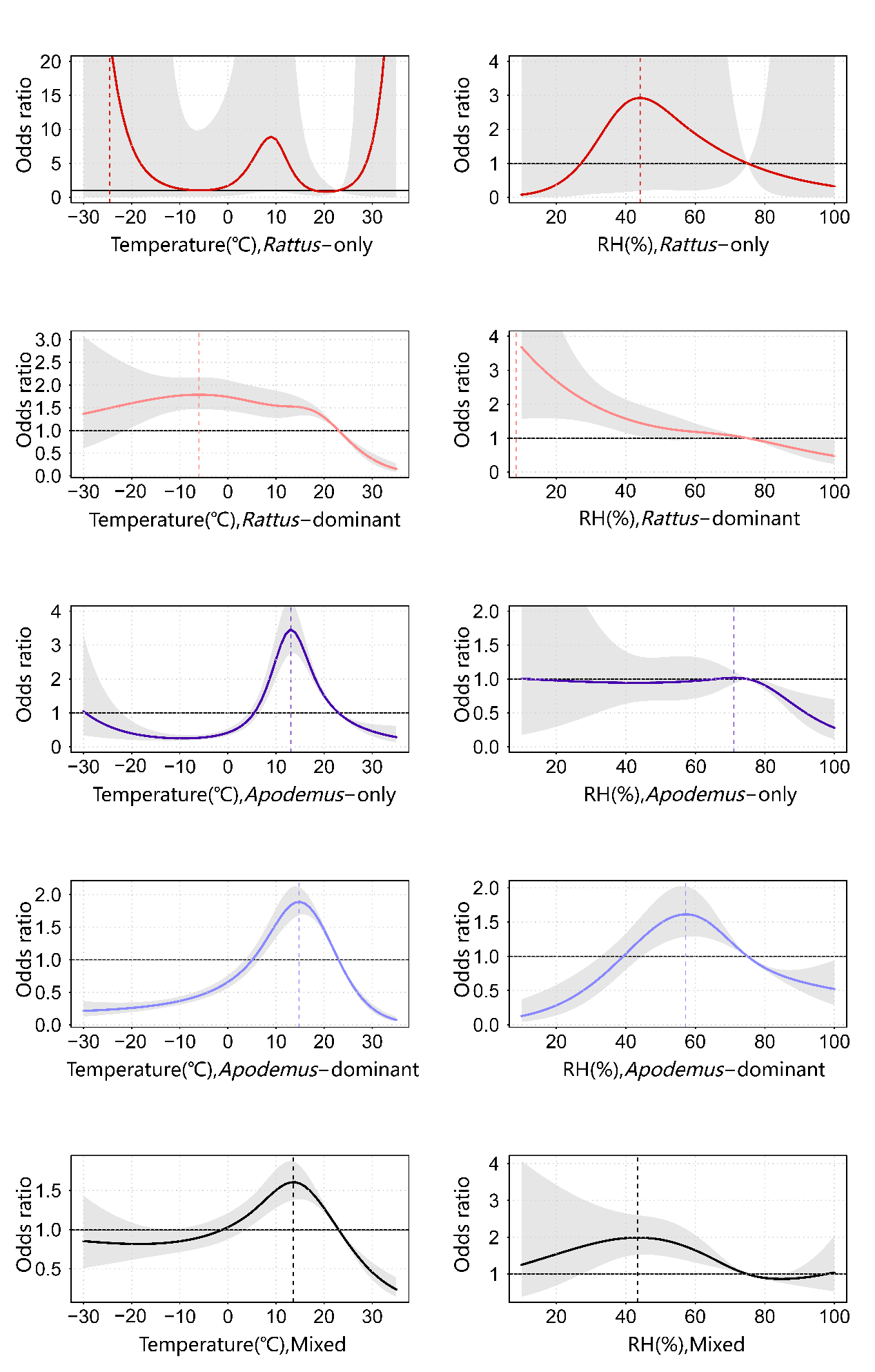
**

**Figure S1** The cumulative odds ratio of HFRS in five types of epidemic areas over the 60 days after the exposure.

Abbreviations: *RH* relative humidity. Notes: The reference point for temperature is 23℃, for relative humidity is 75%.


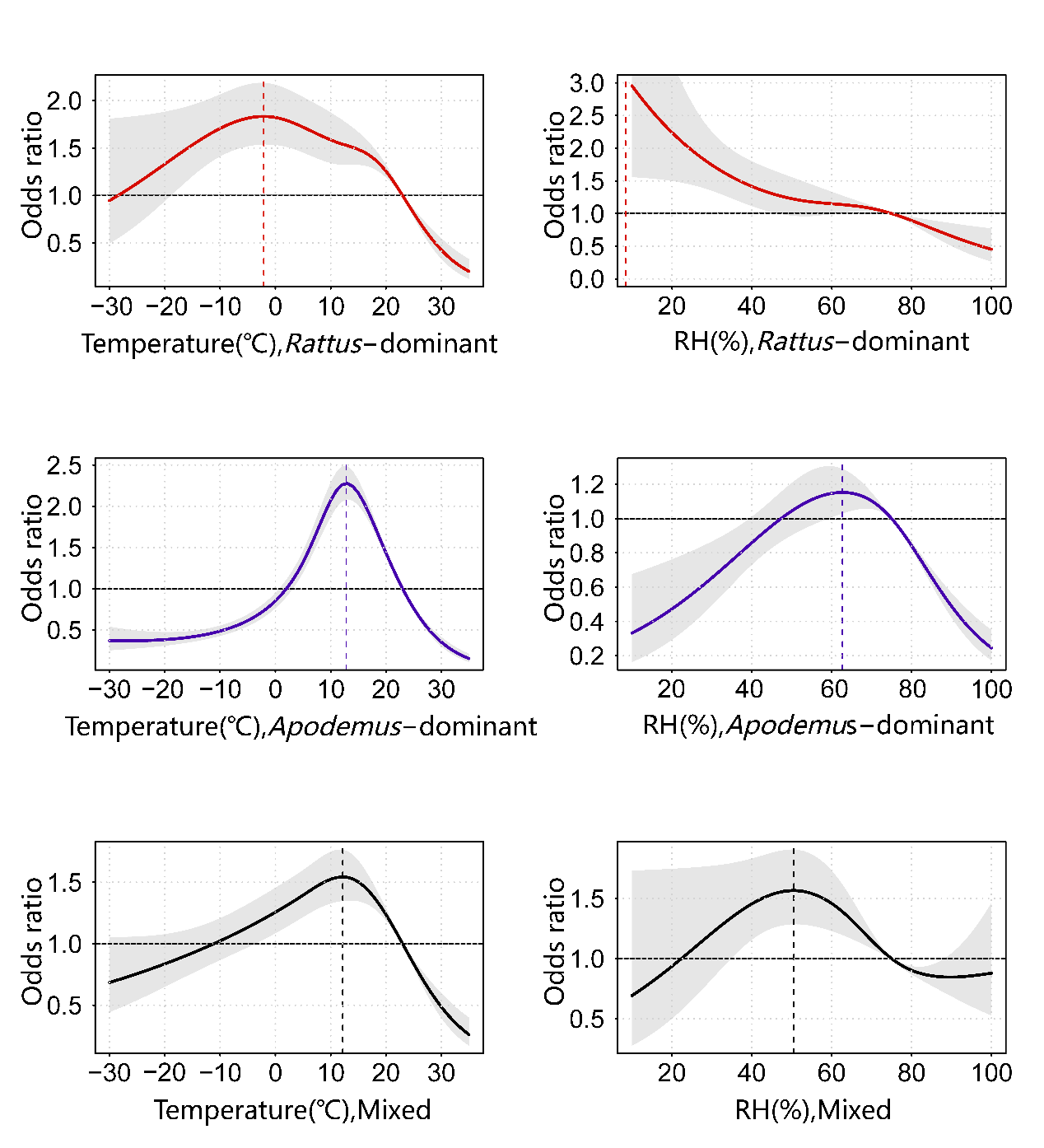


**Figure S2** The cumulative odds ratio of HFRS in three types of epidemic areas over the 45 days after the exposure.

Abbreviations: *RH* relative humidity. Notes: The reference point for temperature is 23℃, for relative humidity is 75%.


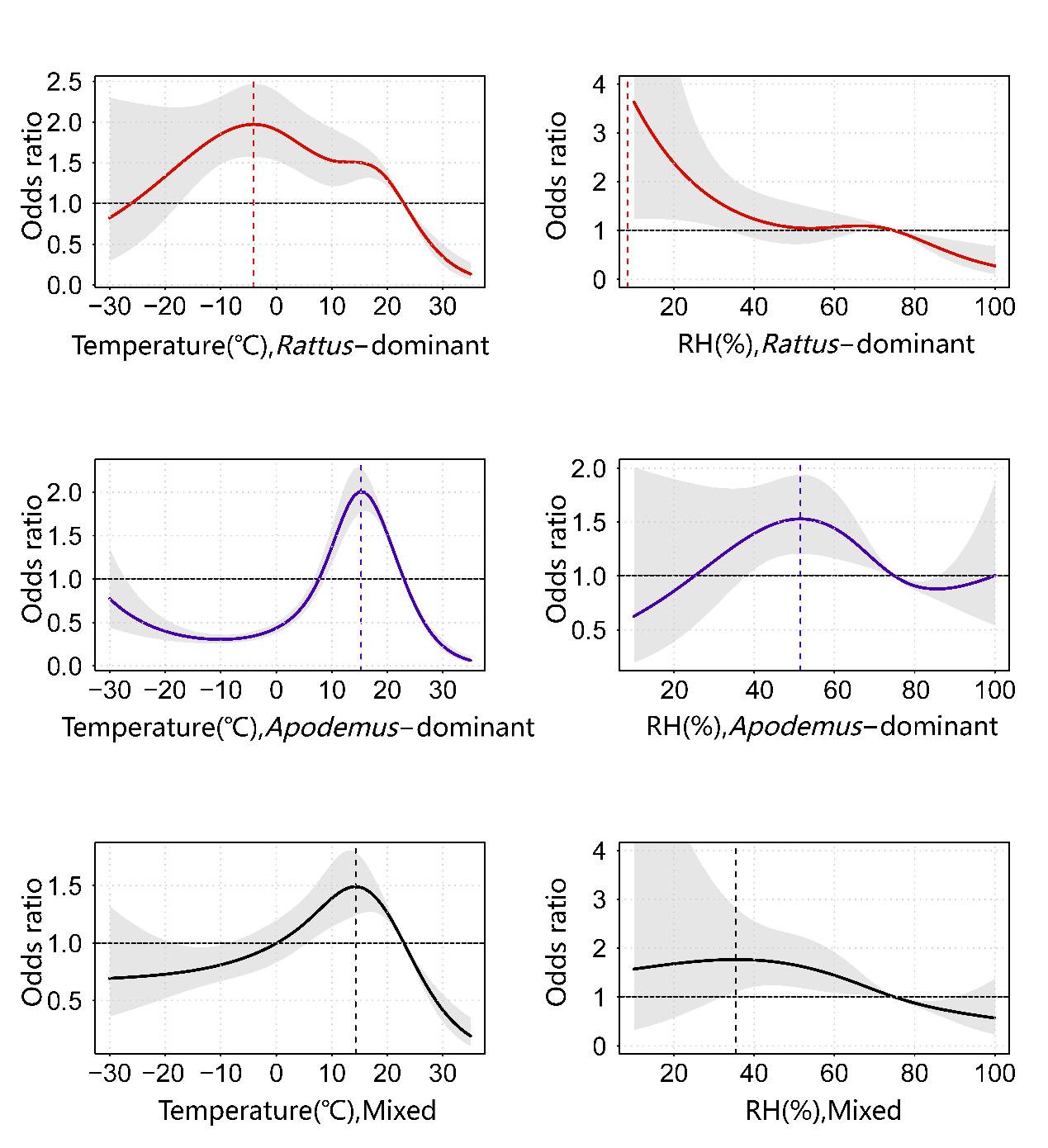


**Figure S3** The cumulative odds ratio of HFRS in three types of epidemic areas over the 75 days after the exposure.

Abbreviations: *RH* relative humidity. Notes: The reference point for temperature is 23℃, for relative humidity is 75%.


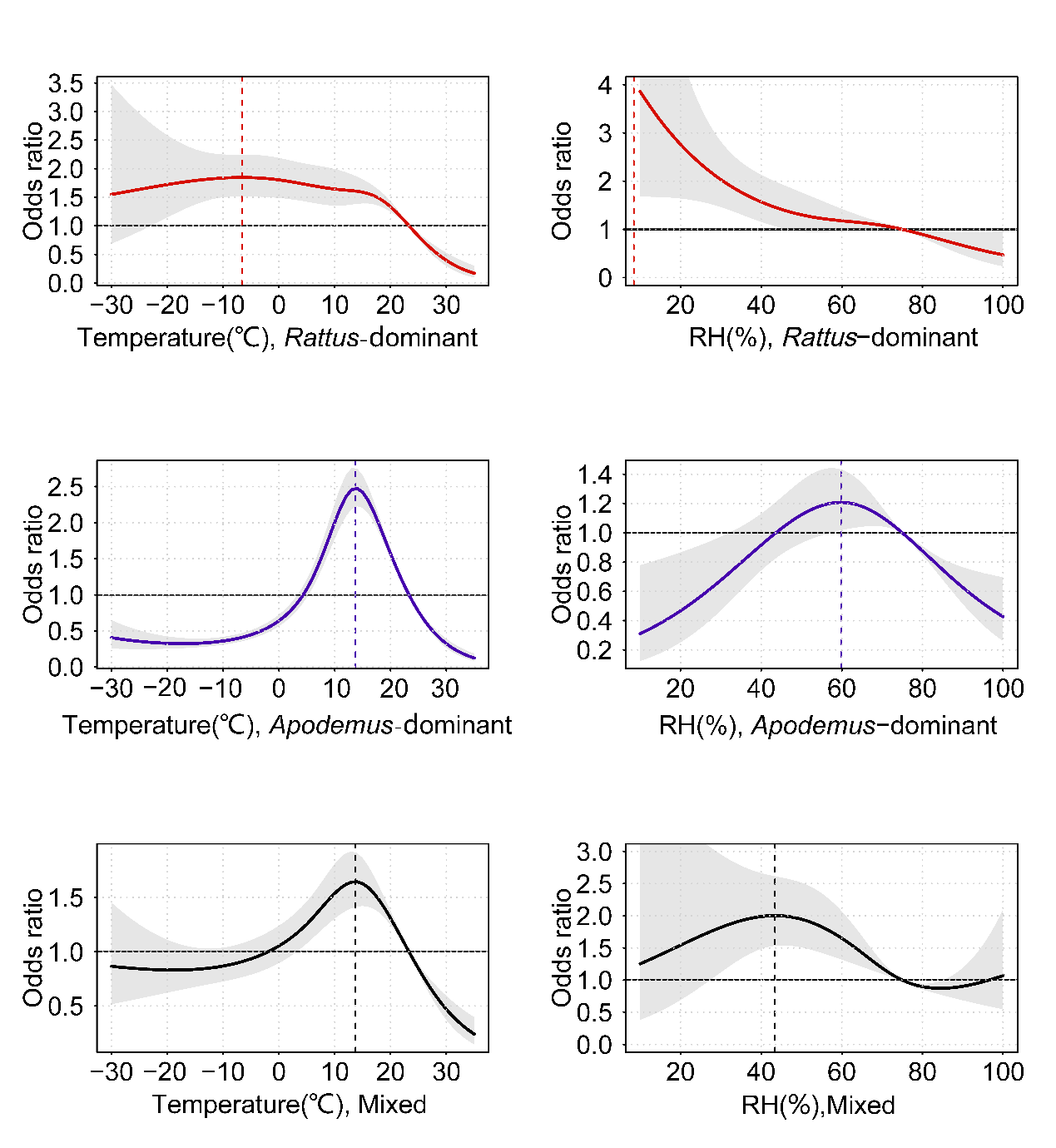


**Figure S4** The cumulative odds ratio of HFRS in three types of epidemic areas over the 60 days after exposure, adjusted for additional meteorological covariates (wind speed, precipitation, and hours of sunshine).

Abbreviations: *RH* relative humidity. Notes: The reference point for temperature is 23℃, for relative humidity is 75%.

**Table S3** Comparison of Literature-Based Classification Results with Study's Classification

| City ID | City | Literature Classification^*^ | Study Classification^*^ |
| --- | --- | --- | --- |
| 110000 | Beijing | A[1, 2] | A |
| 130100 | Shijiazhuang | A[3] | A |
| 130200 | Tangshan | A[4-6] | A |
| 130300 | Qinhuangdao | A[5-9] | A |
| 130500 | Xingtai | A[10] | A |
| 130600 | Baoding | A[11, 12] | A |
| 130700 | Zhangjiakou | A[10] | A |
| 130800 | Chengde | A[4, 6, 13] | A |
| 130900 | Cangzhou | A[11, 14, 15] | A |
| 131100 | Hengshui | A[10] | A |
| 140900 | Xinzhou | A[16] | A |
| 150400 | Chifeng | A[17] | A |
| 150800 | Bayannur | A[18] | A |
| 210100 | Shenyang | A[19-21] | A |
| 210600 | Dandong | B[22] | B |
| 210700 | Jinzhou | A[23, 24] | A |
| 210900 | Fuxin | A[23] | A |
| 211100 | Panjin | A[23] | A |
| 211200 | Tieling | A[23] | A |
| 211300 | Chaoyang | A[23] | A |
| 211400 | Huludao | A[25] | A |
| 220500 | Tonghua | C[26] | C |
| 220800 | Baicheng | A[27] | A |
| 230800 | Jiamusi | C[28] | C |
| 320700 | Lianyungang | B[29] | B |
| 330200 | Ningbo | A[30, 31] | A |
| 330300 | Wenzhou^##^ | A[32] | C |
| 330800 | Quzhou | B[33, 34] | B |
| 331000 | Taizhou | B[35] | B |
| 331100 | Lishui | B[36] | B |
| 341200 | Fuyang | B[37-39] | B |
| 350100 | Fuzhou | A[40] | A |
| 350200 | Xiamen | A[41] | A |
| 350500 | Quanzhou | A[42, 43] | A |
| 350900 | Ningde | C[44] | C |
| 360900 | Yichun | B[45] | B |
| 370100 | Jinan^#^ | A[46]; C[47] | C |
| 370300 | Zibo^#^ | A[48]; B[49]; C[50, 51] | C |
| 370800 | Jining | C[52] | C |
| 429004 | Xiantao^##^ | B[53] | C |
| 429005 | Qianjiang | C[54] | C |
| 430100 | Changsha | C[55] | C |
| 440100 | Guangzhou^#^ | A[56, 57]; C[58] | C |
| 440300 | Shenzhen^##^ | A[59-61] | C |
| 441800 | Qingyuan^##^ | A[62] | C |
| 450100 | Nanning | A[63] | A |
| 510100 | Chengdu | B[64] | B |
| 510700 | Mianyang | B[64] | B |
| 510800 | Guangyuan | B[64] | B |
| 510900 | Suining | B[64] | B |
| 511300 | Nanchong | B[64] | B |
| 511400 | Meishan | B[64] | B |
| 511500 | Yibin | B[64] | B |
| 511600 | Guang'an | B[64] | B |
| 511700 | Dazhou | B[64] | B |
| 511900 | Bazhong | B[64] | B |
| 512000 | Ziyang | B[64] | B |
| 513300 | GarzêTibetanAutonomousPrefecture | B[64] | B |
| 520100 | Guiyang | B[65] | B |
| 530300 | Qujing^##^ | A[66] | C |
| 532300 | ChuxiongYiAutonomousPrefecture | A[67-69] | A |
| 532900 | DaliBaiAutonomousPrefecture | A[68, 70] | A |
| 610100 | Xi'an | B[71-73] | B |
| 610300 | Baoji | B[74] | B |
| 611000 | Shangluo | B[75] | B |
| 640400 | Guyuan | B[76, 77] | B |

^*^A: *Rattus*-dominant area; B: *Apodemus*-dominant area; C: mixed epidemic area

^#^: The classification results of this city are consistent with some literature but inconsistent with others.

^##^: The classification results of this city differ from the literature.

**Reference**

1. Wang RQ, Tang YQ, Li S, Cai X, Liu CC, Jin YY. Analysis of epidemic characteristics of hemorrhagic fever with renal syndrome in Changping District, Beijing. Dis Prev Control Bull. 2021;36(06):34-6. (in Chinese)

2. Cai W, Dun Z, Wang L, Wang BC, Su X, Ding J. Investigation of natural hantavirus infection in rodents in Haidian District, Beijing. Chin J Vector Biol Control. 2009;20(05):470-2. (in Chinese)

3. Guo JH, Zhang BZ, Liu L, Zhang SZ, Zhou JK. Analysis of epidemic characteristics of hemorrhagic fever with renal syndrome in Shijiazhuang, Hebei Province, from 1984 to 2008. Dis Surveill. 2009;24(10):746-9. (in Chinese)

4. Wei YM, Han ZY, Cai YN, Han X, Liu SY, Zhang YB, et al. Analysis of epidemic characteristics of hemorrhagic fever with renal syndrome in Hebei Province, 2018–2019. Chin J Front Health Quar. 2020;43(05):333-6. (in Chinese)

5. Han X, Wei YM, Xu YG, Cai YN, Han ZY, Zhang YB, et al. Analysis of epidemic characteristics of hemorrhagic fever with renal syndrome in Hebei Province, 2013–2015. Chin J Pathog Biol. 2018;13(01):76-8+82. (in Chinese)

6. Cai YN, Han X, Wei YM, Han ZY, Liu SY, Zhang YB, et al. Analysis of epidemic characteristics and host animal surveillance of hemorrhagic fever with renal syndrome in Hebei Province, 2011–2020. Chin J Zoonoses. 2022;38(09):813-7. (in Chinese)

7. Tao X. Surveillance and epidemic analysis of hemorrhagic fever with renal syndrome in Qinhuangdao City, 2013. Occup Health. 2014;30(18):2646-8. (in Chinese)

8. Li ZH. Epidemic analysis and prevention strategies of hemorrhagic fever with renal syndrome in Haigang District, Qinhuangdao, during the first half of 2006. J Med Pest Control. 2007(09):653-4. (in Chinese)

9. Tao X, Peng X, Jia CH, Luo YS, Shi MK, Zheng LX. Surveillance analysis of host animals for hemorrhagic fever with renal syndrome in Qinhuangdao City, 2005–2013. Occup Health. 2015;31(21):2986-8. (in Chinese)

10. Jiang L, Yao MP. Hantavirus transmission model based on epidemic area control measures. Henan Sci. 2017;35(07):1017-21. (in Chinese)

11. Yan H. Establishment of identification methods for common domestic rodent species based on PCR-RFLP technology and host animal surveillance of HFRS in Hebei Province [master's thesis]. 2021. (in Chinese)

12. Gao Y, Zhang HS, Yuan LL, Zhu BG. Analysis of epidemic characteristics of hemorrhagic fever with renal syndrome in Baoding City, 2004–2014. Chin J Pathog Biol. 2016;11(04):349-52. (in Chinese)

13. Chu HN, Zhao GL, Wei F, Li Q, Li QL, Wei SH. Analysis of epidemic characteristics of hemorrhagic fever with renal syndrome in Chengde City, 2009–2014. J Med Pest Control. 2016;32(04):430-2. (in Chinese)

14. Li JJ, Yue CZ, Lu SH, Zhao JX, Li HC, Yang G. Host animal surveillance of hemorrhagic fever with renal syndrome in Xian County, Hebei Province. J Med Pest Control. 2013;29(01):10-2. (in Chinese)

15. Pang ZQ, Pang RX, Lu SH, Qi YM, Zheng ZW. Surveillance analysis of host animals in HFRS epidemic foci in Cangzhou City. J Med Pest Control. 2010;26(06):531+3. (in Chinese)

16. Li JJ, Yue CZ, Lu SH, Zhao JX, Li HC, Yang G. Host animal surveillance of hemorrhagic fever with renal syndrome in Xian County floodplain of Hutuo River. J Med Pest Control. 2013;29(03):251-3. (in Chinese)

17. Zhang LZ, Yin LQ, Li MM, Li BR. Surveillance and epidemic analysis of hemorrhagic fever with renal syndrome in Chifeng City, 2005–2009. J Med Pest Control. 2010;26(12):1158-9. (in Chinese)

18. Gao N, Ma C, Yang PF, Li MH, Zhang YZ. Analysis of epidemic characteristics of hemorrhagic fever with renal syndrome in Bayannur City. China Trop Med. 2008(11):1891-3+9. (in Chinese)

19. Li CT, Wang LY, Jin L. Epidemic analysis of hemorrhagic fever with renal syndrome in Yuhong District, Shenyang, 2004–2007. Med Inform (Mid Edition). 2010;5(02):186-7. (in Chinese)

20. Wang P, Li SQ, Wang ZX. Surveillance and epidemic analysis of hemorrhagic fever with renal syndrome in Shenyang City. Mod Prev Med. 2003(03):422-3. (in Chinese)

21. Che L, Wang XL, Zhang GB, Zhou B. Host animal surveillance analysis of hemorrhagic fever with renal syndrome in Shenyang City, 2011–2020. Chin J Hyg Insect Equip. 2022;28(02):169-73. (in Chinese)

22. Gong RY, Meng XH, Liu Y. Surveillance analysis of hemorrhagic fever with renal syndrome in Dandong City, 2006–2013. China Trop Med. 2014;14(08):1014-5+8. (in Chinese)

23. Zhang J, Liu XS, Wang ZJ, Xu XT, Mao LL, Sun YW. Surveillance analysis of hemorrhagic fever with renal syndrome in Liaoning Province, 2016–2020. Dis Surveill. 2022;37(11):1458-61. (in Chinese)

24. Yang Y, Zhang Z, Zhang X, Bai M. Detection of hantavirus infection rate in rodent lungs by indirect immunofluorescence and RT-PCR. Chin J Hyg Insecticides Equip. 2020;26(01):36-8. (in Chinese)

25. Wang XB, Shen TF, Zhao ZY, Liu B. Analysis of the epidemiological results of hemorrhagic fever with renal syndrome in Huludao City in 2007. Med Anim Control. 2008(07):538-9. (in Chinese)

26. Cui YL, Liu H, Liu YF, Chen XJ, Zhong XH, Zhang ZS. Investigation of host animals of hemorrhagic fever with renal syndrome in Tonghua City. Chin J Hyg Insecticides Equip. 2009;15(02):161-2. (in Chinese)

27. Zheng FQ, Zhang H. Analysis of hemorrhagic fever with renal syndrome epidemic in Baicheng City from 2002 to 2006. Chin Mod Drug Appl. 2007(11):109. (in Chinese)

28. Wang JL, Zhao YS. Analysis of the epidemic characteristics of hemorrhagic fever with renal syndrome in Jiamusi City from 1949 to 2012. Chin J Dis Control. 2015;19(02):211-2. (in Chinese)

29. Hao JH, Zhao. Analysis of the epidemic characteristics of hemorrhagic fever with renal syndrome in Lianyungang City. Mod Prev Med. 2003(06):869-71. (in Chinese)

30. Chen LF, Luo XH, Chen J. Analysis of the monitoring results of hemorrhagic fever with renal syndrome in Yuyao City from 1996 to 2015. Prev Med. 2016;28(07):697-9. (in Chinese)

31. Luo YN. Analysis of the monitoring results of hemorrhagic fever with renal syndrome in Cixi City from 1996 to 2011. Chin J Vector Biol Control. 2012;23(03):265-7. (in Chinese)

32. Lin XD, Guo WP, Wang W, Zhang XH, Chen Y, Xie HB, et al. Epidemiology of hantavirus between humans and animals in Wenzhou City. Chin J Vector Biol Control. 2010;21(03):235-7. (in Chinese)

33. Chen ZB, Lei JB, Lan F. Analysis of the monitoring results of hemorrhagic fever with renal syndrome in Longyou County from 2004 to 2010. Chin J Vector Biol Control. 2011;22(02):178-9. (in Chinese)

34. Wang M, Yang H, Yu ZY, Zhong JY, Fang CF, Wu SQ, et al. Epidemiological characteristics and host animal monitoring of hemorrhagic fever with renal syndrome in Quzhou City, Zhejiang Province, from 2006 to 2020. Chin J Vector Biol Control. 2022;33(04):480-4+509. (in Chinese)

35. Yao PP, Xu F, Zhu HP, Yang ZN, Sun YS, Lu HJ, et al. Investigation and virus isolation of hantavirus host animals in Tiantai County. Prev Med. 2019;31(05):433-6+40. (in Chinese)

36. Yao PP, Lei YL, Xu F, Zhu HP, Mei LL, Wang FS, et al. Isolation and genotyping of hantavirus in Lishui, Zhejiang Province, in 2007. Chin J Zoonoses. 2009;25(02):100-2+6. (in Chinese)

37. Li HB, Sun L, Feng S, Sun QK, Tian YZ, Jiang T, et al. Analysis of the epidemic trend and host animal monitoring of hemorrhagic fever with renal syndrome in Fuyang City. Chin J Hyg Insecticides Equip. 2023;29(04):289-94. (in Chinese)

38. Jian HH, Wan JF, Zhu LY, Jiang T, Tian YZ. Analysis of the monitoring results of hemorrhagic fever with renal syndrome in Fuyang City from 2006 to 2010. Anhui Prev Med. 2012;18(01):31-3. (in Chinese)

39. Du J, Wan JF, Zhu LY, Jiang T, Ding ZT, Liu QQ. Analysis of the monitoring results of hemorrhagic fever with renal syndrome in Fuyang City in 2008. Anhui Med. 2010;31(04):379-81. (in Chinese)

40. Chen Y, Lin DH, Chen L, Lin W, Wang JX, Han TW, et al. Analysis of the 2012 epidemic and host animal monitoring of hemorrhagic fever with renal syndrome in Fujian Province. Chin J Vector Biol Control. 2014;25(02):177-9. (in Chinese)

41. Chen GW, Guo ZN, Huang JW, Chen HF, Xu XR, Chen M. Investigation of host animal infection status of hemorrhagic fever with renal syndrome in Xiamen City. Chin J Hyg Insecticides Equip. 2012;18(04):305-7. (in Chinese)

42. Zheng DF, Chen ZY, Gong CT, Lin MF. Monitoring results of suspected cases of hemorrhagic fever with renal syndrome and host animals in some regions of Quanzhou City. Strait Prev Med J. 2024;30(02):32-5. (in Chinese)

43. Wang YL, Yang YL. Analysis of monitoring results of hemorrhagic fever with renal syndrome in Jinjiang City from 2011 to 2012. Strait Prev Med J. 2013;19(06):41-2. (in Chinese)

44. He S, Chen Y, Lin W, Wang JX, Li SY, Deng YQ. Analysis of the monitoring results of hemorrhagic fever with renal syndrome in Fujian Province in 2010. Strait Prev Med J. 2012;18(03):26-7. (in Chinese)

45. Zhao WD, Wen JS. Analysis and trend prediction of hemorrhagic fever with renal syndrome epidemic in Shanggao County from 1979 to 2009. Chin J Dis Control. 2011;15(12):1084-6. (in Chinese)

46. Xu HR, Xu SH, Sui QM. Analysis of the monitoring results of hemorrhagic fever with renal syndrome in Jinan City from 2002 to 2007. Chin J Prev Med. 2009;10(02):134-7. (in Chinese)

47. Zhao H, Zhao BT, Liu ZW, Zhang QY, Wang L, Jiao HT. Genetic characteristics of hantavirus carried by rodents in hemorrhagic fever epidemic areas in Jinan City. Chin J Prev Med. 2024;25(01):92-6. (in Chinese)

48. Wang L, Zhang Y, Zhang L, Liu FY, Sun T, Jiang XL, et al. Epidemiological characteristics and host animal monitoring of hemorrhagic fever with renal syndrome in Zibo City from 2015 to 2018. Chin J Dis Control. 2020;24(02):237-40. (in Chinese)

49. Wang L, Cui F, Yang SX, Wang ZQ. Analysis of the monitoring results of hemorrhagic fever with renal syndrome in Zibo City from 2006 to 2012. Mod Prev Med. 2014;41(12):2269-71. (in Chinese)

50. Wang L, Liu FY, Jiang XL, Cao HX, Sun T, Zhang L, et al. Analysis of the epidemiological characteristics of hemorrhagic fever with renal syndrome in Zibo City from 2006 to 2019. Mod Prev Med. 2021;48(10):1747-52. (in Chinese)

51. Gong CH, Yuan SZ, Sun MZ. Survey of host animals carrying hantavirus in Zibo area. Med Anim Control. 2002(12):693-5. (in Chinese)

52. Sun QJ, Wang S. Analysis of epidemiological characteristics of hemorrhagic fever with renal syndrome in Rencheng District, Jining City. South China Prev Med. 2005(03):21-4. (in Chinese)

53. Peng Y, Zou WJ, Li GM. Genetic characteristics of hantavirus carried by rodents in Xiantao area, Hubei Province in 2012. J Lab Med Clin. 2015;12(18):2720-1. (in Chinese)

54. Li MC, Hu MJ, Liu JM. Analysis of the epidemiological characteristics of hemorrhagic fever with renal syndrome in Qianjiang City from 2008 to 2017. Public Health Prev Med. 2019;30(02):65-8. (in Chinese)

55. Yu LZ, Fu JR, Wang YP. Monitoring results of hemorrhagic fever with renal syndrome in Ningxiang County from 2006 to 2010. Pract Prev Med. 2012;19(02):207-8. (in Chinese)

56. Liu Y, Lu EJ, Xiong Y, Jiang LY, Xu JX, Wu BJ, et al. Analysis of the monitoring results of hemorrhagic fever with renal syndrome in Guangzhou City in 2005. J Prev Med Inf. 2006(05):536-8. (in Chinese)

57. Li YL, Jing QL, Cao Q, Xu Y, Luo L, Xiao XC. Analysis of the epidemiological characteristics of hemorrhagic fever with renal syndrome in Guangzhou City from 2008 to 2014. Dis Monit. 2015;30(07):595-8. (in Chinese)

58. Qiu JC, Liang X, Luo JB, Pan ZM, Yang ZC, Qiu GX. Analysis of the monitoring results of hemorrhagic fever with renal syndrome in Guangzhou City from 2001 to 2002. Chin Trop Med. 2003(06):745-6. (in Chinese)

59. Liu JJ, Yang F, He JF, Zhang XL, Liang ZN, Zhang SX, et al., editors. Molecular epidemiological study of hantavirus infection in host animals in Shenzhen City. Second Annual Academic Conference of the Chinese Preventive Medicine Association and the Second Annual Conference of the Global Chinese Public Health Association; 2006; Xianghe, Hebei, China. (in Chinese)

60. Cai CL, Chen WS, Chen WH, Zhuo F, Yao XJ, Zhang RL. Serological survey of hantavirus in rodents from hemorrhagic fever epidemic points in Shenzhen area. Chin J Hyg Lab Med. 2017;27(01):127-8. (in Chinese)

61. Yang F, Guli Bahar, Liu JJ, Yang H, Zhang XL, He JF, et al. Rodent surveillance and virus strain SZ2083 isolation and identification of hantavirus infection in rodents in Shenzhen in 2005. Chin J Epidemiol. 2006;11:981-4. (in Chinese)

62. Chen WQ, Luo WL, Huang JY, Li BQ. Investigation on hantavirus infection status in rodents and healthy populations in Qingyuan city. Occup Health. 2018;34(22):3149-51. (in Chinese)

63. Tan Y, Bi FY, Wei ZL, Yang JY. Host animals and virus infection status of hemorrhagic fever with renal syndrome in Guangxi. Chin J Vector Biol Control. 2010;21(06):566-8. (in Chinese)

64. Yuan W, Liu XC, Zhang JK, Lin SH, Chen DL. Analysis of surveillance results for hemorrhagic fever with renal syndrome in Sichuan province from 2007 to 2009. Prev Med Inform. 2010;26(10):776-9. (in Chinese)

65. Luo YX, Jiang L, Liu MQ, Cai XH. Surveillance of host animals for hemorrhagic fever with renal syndrome and epidemic analysis in Guiyang city. Guangxi Prev Med. 2002;05:297-9. (in Chinese)

66. Jin SH, He LF, Zhou JH, Yang WH, Zhang YZ, Feng Y, et al. Survey on hantavirus host animals in Qujing city, Yunnan province. Dis Prev Control Bull. 2013;28(05):4-6. (in Chinese)

67. Gao LF, Wu XL, Hu HM, Luo QM, Yan Y, Qian FB, et al. Epidemiological characteristics of hemorrhagic fever with renal syndrome in Chuxiong Prefecture from 2005 to 2015. Mod Prev Med. 2017;44(04):588-92. (in Chinese)

68. Yang WH, Yang XL, Yang LF, Kuang GP, Li HC, Pan H, et al. Analysis of hemorrhagic fever with renal syndrome cases and host animal survey in Yunnan province in 2020. Chin J Vector Biol Control. 2022;33(03):394-9. (in Chinese)

69. Hu TS, Hu QL, Li SX, Huang Y, Hu HM, Gao LF, et al. Epidemiological characteristics and host animal survey of hemorrhagic fever with renal syndrome in Chuxiong city, Yunnan province from 2015 to 2018. Chin J Vector Biol Control. 2020;31(02):152-7+63. (in Chinese)

70. Li WJ, Zhang HL, Zhang YZ, Yang WH, Zhou JH, Duan CL, et al. Analysis of the epidemiological characteristics of hemorrhagic fever with renal syndrome in Xiangyun county, Yunnan province. Chin Trop Med. 2013;13(11):1319-22. (in Chinese)

71. Jin TZ. Spatiotemporal dynamics analysis of hemorrhagic fever with renal syndrome and host animals in Xi'an city [Master's thesis]. 2015. (in Chinese)

72. Jin TZ, Wu R, Chen HL, Chen XN, Ma CF, Wang KF. Survey on host animals of hemorrhagic fever with renal syndrome and pathogen analysis in Xi'an city. Chin J Vector Biol Control. 2015;26(04):379-82. (in Chinese)

73. Tan X, Xiao D, Yan YP. Analysis of hemorrhagic fever with renal syndrome outbreaks in Huxian county, Xi'an city from 1971 to 2010. Chin J Vector Biol Control. 2012;23(06):577-80. (in Chinese)

74. Li HB, Fu HL, Hu K, Hu XQ, Deng F, Zhang KJ, et al. Monitoring analysis of host animals for hemorrhagic fever with renal syndrome in Baoji city from 2014 to 2018. Chin J Health Insecticides Equip. 2020;26(06):529-32. (in Chinese)

75. Zhang P, Chen YW, Duan YB, He T, Qi YL, Li Q. Monitoring analysis of host animals for hemorrhagic fever with renal syndrome in Shangluo city, Shaanxi province from 2013 to 2015. Med Anim Control. 2020;36(01):63-5+8. (in Chinese)

76. Li T, Li H, Zhan J, Gong R, Li HJ. Monitoring analysis of host animals for hemorrhagic fever with renal syndrome in Guyuan city, Ningxia Hui Autonomous Region from 2015 to 2021. Chin J Vector Biol Control. 2024;35(01):14-20. (in Chinese)

77. Ma XM, Ma JT, Li HJ, Yan Y, Zhan J, Zhang Z. Monitoring analysis of host animals for hemorrhagic fever with renal syndrome in Jingyuan county, Ningxia from 2005 to 2012. J Ningxia Med Univ. 2014;36(02):187-90. (in Chinese)
